# Preliminary guideline for reporting bibliometric reviews of the biomedical literature (BIBLIO): a minimum requirements

**DOI:** 10.1186/s13643-023-02410-2

**Published:** 2023-12-15

**Authors:** Ali Montazeri, Samira Mohammadi, Parisa M.Hesari, Marjan Ghaemi, Hedyeh Riazi, Zahra Sheikhi-Mobarakeh

**Affiliations:** 1 Population Health Research Group, Health Metrics Research Center, Iranian Institute for Health Sciences Research, ACECR, Tehran, Iran; 2 Department of Epidemiology and Biostatistics, Schulich School of Medicine and Dentistry, Western University, London, Canada; 3 Vali-E-Asr Reproductive Health Research Center, Family Health Research Institute, Tehran University of Medical Sciences, Tehran, Iran; 4 Department of Midwifery and Reproductive Health, School of Nursing and Midwifery, Shahid Beheshti University of Medical Sciences, Tehran, Iran; 5 Quality of Life Research Groups, Breast Cancer Research Center, Motamed Cancer Institute, ACECR, Tehran, Iran

**Keywords:** Reporting guideline, Checklist, BIBLIO, Bibliometric reviews, Bibliography

## Abstract

**Background:**

A bibliometric review of the biomedical literature could be essential in synthesizing evidence if thoroughly conducted and documented. Although very similar to review papers in nature, it slightly differs in synthesizing the data when it comes to providing a pile of evidence from different studies into a single document. This paper provides a preliminary guideline for reporting bibliometric reviews of the biomedical literature (BIBLIO).

**Methods:**

The BIBLIO was developed through two major processes: literature review and the consensus process. The BIBLIO started with a comprehensive review of publications on the conduct and reporting of bibliometric studies. The databases searched included PubMed, Scopus, Web of Sciences, and Cochrane Library. The process followed the general recommendations of the EQUATOR Network on how to develop a reporting guideline, of which one fundamental part is a consensus process. A panel of experts was invited to identify additional items and was asked to choose preferred options or suggest another item that should be included in the checklist. Finally, the checklist was completed based on the comments and responses of the panel members in four rounds.

**Results:**

The BIBLIO includes 20 items as follows: title (2 items), abstract (1 item), introduction/background (2 items), methods (7 items), results (4 items), discussion (4 items). These should be described as a minimum requirements in reporting a bibliometric review.

**Conclusions:**

The BIBLIO for the first time provides a preliminary guideline of its own kind. It is hoped that it could contribute to the transparent reporting of bibliometric reviews. The quality and utility of BIBILO remain to be investigated further.

## Background

Several guidelines exist for reporting findings of different study designs. The detailed explanations and checklists for such guidelines can be found in Enhancing the Quality and Transparency of Health Research (EQUATOR) Network [1] and are available to research communities [2]. For instance, the quality of reporting of meta-analyses (QUOROM) statement for improving the quality of reporting meta-analyses of randomized controlled trials was first published in *The Lancet* in 1999 [3]. Consequently, the work was further improved, and it was replaced with the preferred reporting items for systematic reviews and meta-analyses (PRISMA) [4]. This guideline was published simultaneously in 6 journals in 2009 [4–9], and since then, many biomedical journals and investigators have adhered to this instruction. The instruction also was extended, and complementary versions of the guideline either are developed (such as PRISMA for Abstracts) [10] or are under development (e.g., PRISMA for children) [11]. Even the preferred reporting items for overviews of reviews (PRIOR) are proposed [12], and a recent call by *Systematic Reviews* (the journal) indicates that attempts to enhance the knowledge of this type of reporting are in progress [12, 13].

**Fig. 1:**
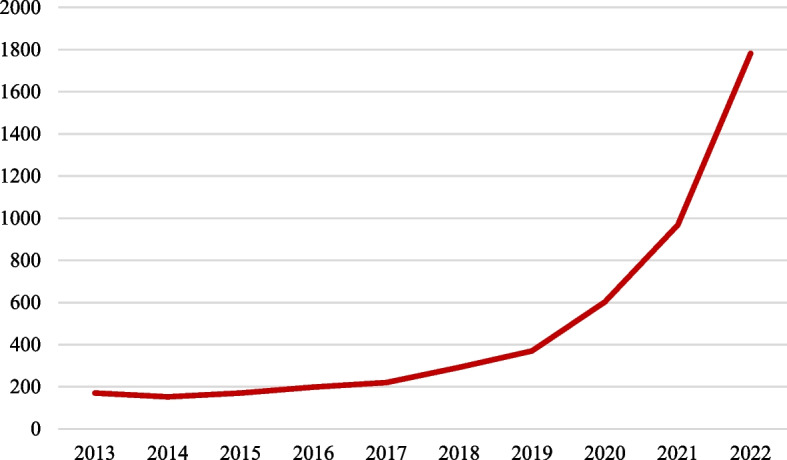
Papers with bibliography/bibliographic and bibliometric in the title of publications during 2013–2022 (PubMed)

However, we believe there is also a need for a guideline for another type of reporting, namely, Guideline for Reporting Bibliometric Reviews of the Biomedical Literature (BIBLIO). A bibliometric or a bibliographic review of the literature is different from an overview. Recently, the literature witnessed a relatively considerable number of bibliometric analyses of the biomedical literature [14–23]. The number of publications related to various topics with bibliometric or bibliography/bibliographic in the title during the last 10 years is presented in Fig. 1. Therefore, this paper attempts to propose a preliminary version of a guideline for reporting bibliometric reviews of the literature. The guideline was developed based on all existing guidelines presented in the EQUATOR Network [1]. In addition, experiences from writing a number of bibliometric reviews [24–28] helped the authors to formulate this first version of the work with the courage that it could be improved further by receiving feedbacks from other scholars in the field.

Although BIBLIO is in its preliminary stage of development and there is no evidence of its quality and utility, it is hoped that it could contribute to the transparent reporting of bibliometric reviews. The application of bibliometric reviews enables one to analyze vast amounts of publications and their production patterns on macroscopic and microscopic levels [29]. Therefore, this study aimed to provide a guideline for reporting bibliometric reviews. The BIBLIO checklist was registered in the EQUATOR Network on 19 October 2021 [30].

### History

**Fig. 2:**
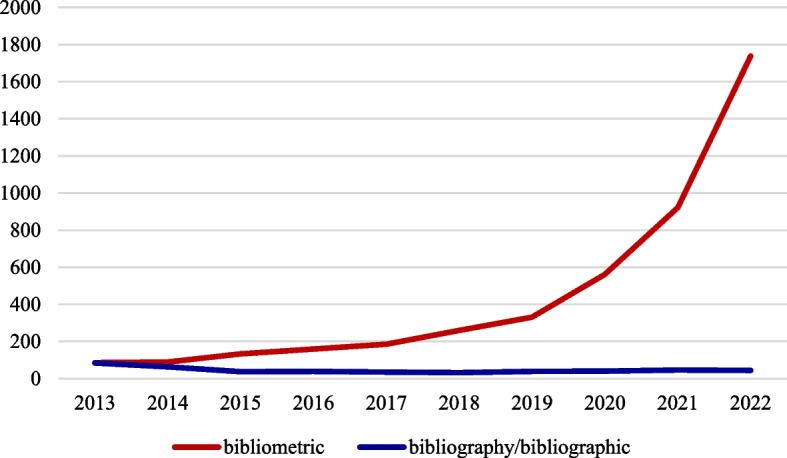
Trends of using bibliography/bibliographic or bibliometric in the title of publications during 2013–2022 (PubMed)

The term bibliometric and bibliography are used interchangeably in the literature. Earlier, the term bibliography was more popular, but it was gradually replaced with the bibliometric expression (Fig. 2). The history of the statistical bibliography as reviewed by Thackray [31] indicates that the root goes back to early 1900s as this was acknowledged in a paper by Garfield [32] and a number of scholar such as Cole and Eames (1917), Hulme (1923), Lotka (1926), and Gross and Gross (1927) were listed as those who contributed to the technic of statistical analysis of the literature. However, it was Otlet in 1934 who first used the term “bibliometrie” and defined it as “the measurement of all aspects related to the publication and reading of books and documents” [33, 34]. Then in 1969, Pritchard coined the term “bibliometrics” and defined it as “all the studies which seek to quantify the processes of written communication” [35]. The detailed history since 1934 is presented in Table 1.

**Table 1:** A chronological list of definitions of bibliometrics, based on the historical development of the term and its definitions

Author(s)	Year	Definition
Otlet [33, 34]	1934	The measurement of all aspects related to the publication and reading of books and documents
Pritchard [35]	1969	The application of mathematics and statistical methods to books and other media of communication
Fairthorne^a^	1969	Quantitative treatment of the properties of recorded discourse and behavior appertaining to it
Donohue^a^	1972	Quantitative analysis of gross bibliographical units such as books, journal article’s, and the like
Hawkins^a^	1977	Quantitative analyses of the bibliographic features of a body of literature
Nicholas and Ritchie^a^	1978	Bibliometrics is the statistical or quantitative description of a literature—“literature” taken here to mean, simply, a group of related documents
Potter^a^	1981	Bibliometrics is, simply put, the study and measurement of the publication patterns of all forms of written communication and their authors
Schrader^a^	1981	The scientific study of recorded discourse
Machlup and Mansfield^a^	1983	Statistical studies of the growth and distribution of the literature (e.g., the area known as bibliometrics)
ALA Glossary of Library and Information Science^a^	1983	The use of statistical methods in the analysis of a body of literature to reveal the historical development of subject fields and patterns of authorship, publication, and use
Harrod’s Librarians Glossary^a^	1984	The study of the use made of books and other media within and between library systems
Boyce and Kraft^a^	1985	Bibliometrics is the quantitative study of written communication through its physical realization
De Glas^a^	1986	Generally speaking bibliometrics could be defined as the search for systematic patterns in comprehensive bodies of literature
Meanwhile, Garfield, Malin and Smal^a^	1987	Bibliometrics can be defined as the quantification of bibliographic information for use in analysis
Hawkins [36]	2001	The quantitative analysis of the bibliographic features of a body of literature
De Bellis [37]	2009	Analyze, quantify, and measure communication phenomena, to build accurate formal representations of their behavior for explanatory, evaluative, and administrative purposes

^a^Derived from [38], otherwise the citation could be find in the reference list. Tabulation was designed by the authors

### Definition

Bibliometric is a type of review that can be used to look at different and important areas of investigations and obtain a general synopsis of published literature [39]. This guideline defines a bibliometric review as follows “a review of all full published papers that appear in the biomedical journals and includes all types of evidence such as descriptive studies, observational studies, experimental studies, qualitative studies, and systematic reviews in order to account for every single evidence exist. The bibliometric of the literature does not include electronic publications a head of print since the ultimate date for such publications are not known”. This definition was formulated based on chronological account of the term bibliometric and its developments [31–38].

### Similarities and differences between systematic reviews and bibliometrics

Bibliometric is similar to systematic review in retrieving the literature [40], but they have low agreement rate regarding relevant literature and the purpose. While systematic reviews are seeking to respond to a very clear question based on good quality evidences, bibliometrics is rather a numeration of evidence without quality assessment. Bibliometrics often rely on the interpretation of quantitative details of publications such as main topics, authors, sources, most impactful authors, most impactful articles, and countries in a particular area in the existing literature. In this type of study, mapping techniques including graphical representations, tabulated forms, network diagrams, and so on are used to present results usually performing these with the assistance of softwares [39–42].

### Development of BIBLIO

The BIBLIO was developed through two major processes: literature review and the consensus process. These are briefly described as follows:

- 1. Literature review for item selection
  The BIBLIO started with a comprehensive review to identify potential items for including in this guideline. The databases searched included PubMed, Scopus, Web of Sciences, and Cochrane Library. The aim was to examine and review all methodological papers on the conduct and reporting of bibliometric studies up to 2021. The search was updated in January 2022 and once during the process of revisions in September 2023. Papers were retrieved using different keywords and MeSH terms including “bibliometric,” “bibliography,” and “bibliographic” in the title of papers. All potentially relevant publications were extracted and reviewed independently by two authors (AM and SM). Overall, 13,720 papers were identified. After removing duplicates and irrelevant documents, only 19 papers [40, 43–60] were found that were dealing with methodological issues. Also, we visited all reporting guidelines for review studies that are indexed in the EQUATOR [1]. The items derived from the literature are shown in Table 2.
- 2. Consensus process
  The process followed the general recommendations of the EQUATOR Network on how to develop a reporting guideline, of which one fundamental part is a consensus process [1]. We used Delphi consensus to obtain advice on how to report a “bibliometrics.” Delphi was performed based on the conducting and reporting Delphi studies (CREDES) guideline [61]. A panel consisted of eleven experts, including bibliometrician, epidemiologist, clinician, librarian, statistician, journal editor, and a research fellow. They were invited to see the list of items derived from the previous stage and asked to identify additional items and to choose preferred options or suggest other items that should be included in the checklist. In each round of the Delphi, the feedback process allowed and encouraged the selected participants to review and assess their own initial judgments. Thus, the results of previous iterations regarding specific items were changed or modified by each member of the expert panel in later iterations based on the review and assessing the comments and feedback provided by the other Delphi panelists [62].

**Table 2:** Items provided from the literature review

	**Item**
**Title**	Identification
	Issues/topics/coverage of time period
**Abstract**	Structured summary
**Introduction/background**	Justification/rationale/explanation
	Objectives
**Methods**	Search engines (data sources)
	Search strategy
	Time period
	Eligibility criteria
	Data refinement (data selection procedure)
	Quality assessment
	Data synthesis
**Results**	Descriptive findings (statistics)
	Schematic map and trend
	Presentation approach
	Synthesis of findings
**Discussion**	Summary of evidence
	Interpretation
	Strengths and limitations
	Conclusion(s)

In the first round of the Delphi process, we used an open-ended questionnaire to solicit specific information and to add suggested items to the list of items and increase the rich of data collection. After receiving the experts’ responses, we converted the collected information into a well-structured questionnaire on a five-point scale with content analysis technique. This questionnaire was used as the survey questionnaire for the second round of data collection. Each Delphi participant received a second questionnaire and was asked to review the items summarized based on the information provided in the first round. Accordingly, we asked Delphi panelists to rate items and state the rationale concerning rating priorities. In the third round, each Delphi panelist received a questionnaire that included the items and ratings summarized in the previous round and was asked to revise their judgments. The remaining items, ratings, minority opinions, and items achieved consensus were distributed to the panelists in the final round. The fourth round provided a final opportunity for participants to revise their responses after formal feedback of the group. At last, the checklist was finalized based on the comments and answers of the panel members in four rounds. The cut-off for consensus was determined by percentage of agreement (mainly 75 to 80%). The duration of each round of Delphi was about 8 weeks, and the length of the overall study process was 8 months. Before beginning the Delphi survey, all experts were asked to disclose any conflicts of interest. The response rate was 100% for all four rounds of the Delphi process.

### Scope of the guideline

BIBLIO is for use in reporting bibliometric reviews and has been designed primarily for bibliometric reviews that evaluate published papers irrespective of the design of the studies. The BIBLIO items are relevant for all types of quantitative and qualitative studies. BIBLIO can be used for reporting original bibliometric reviews and updated bibliometric reviews. BIBLIO is not to guide a bibliometric review conduct. However, familiarity with BIBLIO is helpful when planning and conducting bibliometric reviews to ensure that all recommended information is captured.

### The BIBLIO checklist

The development team provided a list of items based on the literature review and presented them into the consensus process. Participants made revisions to the phrasing and format of the checklist by consolidating and eliminating items during the consensus process. Eventually, the BIBLIO checklist consisted of 20 items that should be described as a minimum requirements in reporting a bibliometric review as follows: title (2 items), abstract (1 item), introduction/background (2 items), methods (7 items), results (4 items), discussion (4 items). The full description of the items is in progress and will be available in due course. However, as an example here, we elaborate on item 15. As shown item 15 provides guidelines for reporting the results. As such four options are proposed. In the following section, we describe each option ensuring that examples given could help investigators to better summarize the findings. Since the opening part of each option is the same here the focus is on how organize the main findings:

- Option 1: Organization based on study design and main study types
  Research design is a blueprint of a scientific study. We could summarize studies based on different designs and main study types. For instance, one might summarize main study types based on randomized trials, observational studies, study protocols, diagnostic/prognostic studies, case reports, clinical practice guidelines, and qualitative studies on a given topic.
- Option 2: Organization based on outcome measures
  The other suggestive way to summarize the main findings is based on outcomes. For instance, a bibliometric analysis that evaluated the impact of race on postoperative outcomes and complications following elective spine surgery was classified based on outcomes providing four categories including general complications, medical complications, surgical complications, and postoperative outcomes [63].
- Option 3: Organization based on concept
  To simplify and clarify this presentation approach, we explain this option with an example. A study on bibliometric analysis of health literacy instruments summarized the findings in four tables according to the concept behind instruments including general instruments, condition-specific health literacy instruments (disease and content), population-specific instruments, and electronic health [28]. Authors could invent such concepts or use the literature for categorizing and summarizing the findings.
- Option 4: Organization based on different subtitles relevant to the main topic
  This presentation approach is well known and was used in many studies. One example for this option is a bibliometric study on health-related quality of life in breast cancer patients. In this study, the findings were summarized and presented according to treatments modalities and a number of classifications including surgical treatment, systemic therapies, psychological distress, supportive care, and common symptoms [26]. One should note there are many ways that we could summarize and tabulate the findings to provide a quick and at the same time a comprehensive perspectives of the studies under review. The checklist is presented in Table 3.

**Table 3:** The BIBLIO checklist for reporting the bibliometric reviews of the biomedical literature

Section/topic	Item no	Checklist item	Reported on page no
**Title**			
Identification	1	Identify the report as a bibliometric review in the title	
Issues/topics	2	Indicate the key issues/topics under investigation and coverage of time period	
**Abstract**			
Structured summary	3	Structured summary including (as applicable): background, methods, results (key findings), and conclusions	
**Introduction/background**			
Justification/rationale/explanation	4	Present review of existing knowledge and epidemiological information	
Objectives	5	Statement of the objective(s) or question(s)	
**Methods**			
Search engines (data sources)	6	Describe all information sources (such as electronic databases, contact with study authors, trial registers, or other gray literature sources)	
Search strategy	7	Keywords and systematization criteria (date of search, language, type of document) for the search	
Time period	8	The period that the review covers and the justification	
Eligibility criteria	9	Describe all inclusion and exclusion criteria, languages, study design, type of publication, and time period	
Data refinement (data selection procedure)	10	Remove the irrelevant articles; inspection to eliminate duplicate and unrelated articles (after evaluation of the title, abstract, and content)	
Quality assessment (optional)	11	Assessment of papers by three authors and the use of assessing checklists	
Data synthesis	12	Describe the methods used for summarizing, handling, synthesis, tabulations, or schematic displays. Describe how the data were analyzed	
**Results**			
Descriptive findings (statistics)	13	- Provide details of the search and selection process in a flow diagram - Number of citations retrieved (number of publication, year of publication, type of documents, country of publication, articles with the highest impact, most impactful authors, most impactful articles, authors with the highest production, top journals, top institutions, …)	
Schematic map and trend	14	Summarize and/or present the schematic maps and trends using an appropriate software to present citations, journals, authors, top journals, time trends, emerging literature, and any relevant indicators (as applicable) [64–68]	
Tabulation and summarizing the findings	15	General recommendation: Studies under consideration could be summarized and organized by different subtitles and different scenarios. Regardless, results need to be presented in separate tables covering each subtitle. The followings are some options that could help to summarize the findings Option 1: - Start the presentation with a historical view [when and who first published on the topic] - Report on review papers. The result should be listed in a separate table. Also, specify the review type (scoping review, narrative review, systematic review, and meta-analysis) - Summarize the findings according to the study designs and main study types Option 2: - Start the presentation with a historical view [when and who first published on the topic] - Report on review papers. The result should be listed in a separate table. Also, indicate the review type (scoping review, narrative review, systematic review, and meta-analysis) should be specified - Summarize the findings according to outcome measures or populations. For example, see [63] Option 3: - Start the presentation with a historical view [when and who first published on the topic] - Report on review papers. The result should be listed in a separate table. Also, specify the review type (scoping review, narrative review, systematic review, and meta-analysis) - Summarize the findings according to concept [28] Option 4: - Start the presentation with a historical view [when and who first published on the topic] - Report on review papers. The result should be listed in a separate table, and also specify the review type (scoping review, narrative review, systematic review, and meta-analysis) - Summarize the findings according to different subtitles relevant to the main topic [26]	
Synthesis of findings	16	Synthesize the findings as much as possible, find the gap, and propose a model, hypothesis, etc. (if applicable)	
**Discussion**			
Summary of evidence	17	Summarize the main findings. The findings should be presented in more “general” or “accessible” terms	
Interpretation	18	Include interpretation consistent with results. Explanations for observed outcomes, similarities, and differences reported would be essential	
Strengths and limitations	19	Discuss the strengths and limitations	
Conclusion(s)	20	Provide a general interpretation of the results with respect to the review questions and objectives, as well as potential implications	

## Discussion

A bibliometric review is a helpful means for accurately and reliably summarizing the evidence, specifically when a large number of papers exist on a given topic [69]. The bibliometric studies that are well done usually could help to grasp the current literature, identify knowledge gaps, derive novel ideas for investigation, and position their intended contributions to the field [43].

The bibliometric methods are quantitative and descriptive by nature but also used to make pronouncements about qualitative aspects. The principal purpose of bibliometric studies is to change intangible knowledge (scientific quality) into manageable entities [70]. Bibliometrics are not in-depth and evaluative reviews. However, they could briefly report on effectiveness and evaluations. Overall, a good bibliometric review should provide a take-home message for its readers.

A number of recommendations are proposed to improve readability of bibliometric reviews. For instance, it was proposed using easy-to-interpret metrics, as non-experts have a difficulty understanding of complex indicators. Also, it was recommended to avoid inventing the indicators, especially composite metrics that mix several indicators in a single measure. Likewise, it was suggested to avoid conscious efforts to manipulate the findings, for instance, choosing metrics that may favor your institution, certain areas, or researchers within it [44].

A bibliometric review could reveal how much effort has been made into a specific topic. In addition, presenting and summarizing the studies allows scholars to use bibliometric analysis to uncover emerging trends in article publishing, journals’ performances, collaboration patterns, and exploring the intellectual structure of a specific domain in the extant literature [71, 72]. Describing the evidence could help policymakers, managers, and other decision-makers to formulate appropriate recommendations for practice or policy [73] and help editors judge the merits of publishing reports of new studies [74]. The bibliometric also helps translate and map the cumulative scientific knowledge and evolutionary nuances of well-established fields by making sense of large volumes of unstructured data in rigorous ways [43].

The use of BIBLIO similar to other guidelines [3, 4, 75] has the potential to benefit many stakeholders. The BIBLIO provides readers with a complete understanding of evidence about the necessity of each item. We have attempted to ensure that the guideline is helpful to authors seeking guidance on what to include in a bibliometric review. We hope the BIBLIO will help increase the quality of reported and published bibliometric reviews. Peer reviewers, editors, and other interested readers might also find the BIBLIO helpful in assessing such reviews. We hope journal editors will encourage authors to include the BIBLIO checklist when submitting a bibliometric review for publication.

Finally, although we followed the general recommendations of the EQUATOR Network and used a literature review and a Delphi consensus process to develop the BIBLIO checklist, it seems that its main limitation is the fact that there is no evidence to suggest it will improve the quality of bibliometric reviews. In this regard, feedback from editors and researchers about details and overall structure can be helpful. Additionally, one should note that bibliometric reviews is not an in-depth review of the literature and rather the most important contribution of this type of reviews is to collect and summarize evidence when we witness a pile of evidence on a topic. As such it reveals that how much effort has been conducted on a topic. In addition, this approach might help investigators to create new questions to conduct more focused studies on the topic in the future [26].

## Conclusion

The BIBLIO provides a reporting guideline for bibliometric reviews of the biomedical literature. We hope that the guideline could result in more transparent and accurate reporting of bibliometric reviews.

## Data Availability

Not applicable.

## Abbreviations

EQUATOR: Enhancing the Quality and Transparency of Health Research
PRISMA: Preferred reporting items for systematic reviews and meta-analyses
PRIOR: Preferred reporting items for overviews of reviews
BIBLIO: Guideline for reporting bibliometric review of the biomedical literature
